# Supplementary material for: Influence of inter-fractional respiratory motion changes on dose delivery accuracy in dynamic conformal arc lung stereotactic body radiotherapy: A phantom study
Source: PLoS One. 2025 Dec 4;20(12):e0336475. doi: 10.1371/journal.pone.0336475 (PMC12677502; doi:10.1371/journal.pone.0336475)
Supplement: S1 Table — (DOCX) [file pone.0336475.s001.docx]

**S1 table. Glass dosimeter calibration**

| Glass dosimeter | Absorbed dose  (cGy) | Exposure dose  (cGy) | Background  (cGy) | Calibration factor |
| --- | --- | --- | --- | --- |
| 1 | 421.0 | 518.1 | 0.027 | 1.2306492 |
| 2 | 424.5 | 518.1 | 0.025 | 1.2205019 |
| 3 | 413.3 | 518.1 | 0.026 | 1.2535767 |
| 4 | 426.0 | 518.1 | 0.027 | 1.2162049 |
| 5 | 422.0 | 518.1 | 0.029 | 1.2277336 |
| 6 | 421.2 | 518.1 | 0.029 | 1.2300654 |
| 7 | 424.3 | 518.1 | 0.030 | 1.2210786 |
| 8 | 420.0 | 518.1 | 0.026 | 1.2335791 |
| 9 | 415.1 | 518.1 | 0.029 | 1.2481417 |
| 10 | 413.8 | 518.1 | 0.028 | 1.2520626 |
| 11 | 410.8 | 518.1 | 0.026 | 1.2612056 |
| 12 | 415.1 | 518.1 | 0.025 | 1.2481405 |
| 13 | 424.8 | 518.1 | 0.025 | 1.2196399 |
| 14 | 402.5 | 518.1 | 0.030 | 1.2872146 |
| 15 | 428.1 | 518.1 | 0.024 | 1.2102380 |
| 16 | 422.6 | 518.1 | 0.029 | 1.2259904 |
| 17 | 422.6 | 518.1 | 0.023 | 1.2259887 |
| 18 | 425.9 | 518.1 | 0.025 | 1.2164899 |
| 19 | 423.8 | 518.1 | 0.025 | 1.2225178 |
| 20 | 420.1 | 518.1 | 0.026 | 1.2332854 |
