# Supplementary material for: Influence of inter-fractional respiratory motion changes on dose delivery accuracy in dynamic conformal arc lung stereotactic body radiotherapy: A phantom study
Source: PLoS One. 2025 Dec 4;20(12):e0336475. doi: 10.1371/journal.pone.0336475 (PMC12677502; doi:10.1371/journal.pone.0336475)
Supplement: Attachment — Submitted filename: Response to reviewers.docx [file pone.0336475.s003.docx]

**RESPONSES TO THE REVIEWERS’ COMMENTS**

Dear Editors and Reviewers,

We thank the editors and reviewers for their careful review of our manuscript with valuable comments and suggestions. A substantial revision of the manuscript has been conducted to take all of them into account. We have done our best to address all the points raised. We believe the manuscript has been significantly improved and hope that it is suitable for consideration for the publication. Each comment is followed by the corresponding answer highlighted in red. In addition, we made corrections and clarifications as the editor and reviewers suggested, all of them are highlighted with ‘track changes’ feature in Microsoft Word within the revised manuscript.

**REVIEWER’S COMMENTS**

**Reviewer #1:**

The study addresses a clinically important question about motion management in lung SBRT, specifically examining the effects of respiratory rate and amplitude on dose delivery using a 4DCT phantom and direct dosimetric measurements.

The authors should address the following questions to further strengthen the manuscript:

1. While direct measurement with a glass dosimeter adds robustness, please provide more detail on the calibration, linearity, energy dependence, and measurement uncertainties. Additionally, please clarify the readout process for the glass dosimeters, including how potential signal fading, background correction, or inter-dosimeter variability were addressed. Thank you for describing the dosimeter placements at the center and edges of the PTV if possible, please elaborate on whether these locations correspond to regions of highest dose gradient or areas most at risk of underdosing in clinical SBRT.

Thank you for your comment. We have revised the Methods section to further clarify the calibration, readout, and uncertainty management of the glass dosimeters.

Regarding dosimeter placement, the dosimeters were positioned in the center, and superior and inferior edges of target. The place is immediately adjacent to the high dose gradient region which makes it sensitive for detecting dosimetric changes caused by motion variations. Any outward shift of the target would cause these specific locations to experience a sharp dose decrease first, allowing us to directly measure the dose in the areas most vulnerable to motion-induced underdosing.

Line (86-104)

All glass dosimeters were annealed prior to irradiation using a small electric oven (Hi-Cera Kiln NHK-210-BS2, Nitto Kagaku, Japan) at 400 °C for 60 min, followed by cooling to below 40 °C for initialization. Standard calibration of the reader was performed using a manufacturer-supplied reference glass element. Calibration factors were determined individually for each glass element under the same static SBRT beam conditions as the experiment, thereby minimizing potential uncertainties such as angular dependence. The reproducibility of calibration factors was verified by repeated irradiations across a wide dose range, confirming the excellent linearity of glass dosimeter response. The energy dependence of glass dosimeters is known to be minimal in the MV photon range, and since all irradiations in this study were performed with a single 6 MV FFF beam, energy dependence was not considered a significant factor.

Before irradiation, the initial background signal of each element (typically 10–30 µGy) was measured and subtracted from subsequent readouts. After irradiation, all elements were pre-heated at 70 °C for 30 min using a forced convection oven (DKN302, Yamato, Japan) to stabilize the radio-photoluminescence signal and minimize fading. Inter-dosimeter variability was managed by applying element-specific calibration factors rather than pooled calibration. All readout data were stored electronically, and the dosimeters were kept in a desiccator to prevent variability due to humidityBefore measurement, the glass dosimeters were annealed at 400℃ for 1 h to enhance the formation of radio-photoluminescent centers. Following irradiation with a 6 MV photon beam, the dosimeters were preheated to 70℃ for 30 min to stabilize the luminescence signal [22].

Line (113-119)

Three dosimeters were placed inside the tumor target to assess dose delivery vulnerabilities to respiratory motion. One dosimeter was placed in the geometric center to verify the planned target dose. Two dosimeters were placed on just inside the superior and inferior edges of the target. While these locations are intended to receive a stable, high prescription dose under ideal conditions, their proximity to the steep dose fall-off region makes them the most sensitive indicators of underdosing resulting from geometric misses due to respiratory motion (Fig 1b).

2. Given that the ITV was generated from the full phase, while the gated PTV used only the 30–70% phase range, could the authors discuss how this difference in phase selection may affect the comparability of the two approaches?

Thank you for your comment. As you pointed out, the ITV and gated SBRT plans were generated based on different respiratory phases. This methodological difference is an essential element of our study, as it reflects the fundamental difference between these two clinical strategies for motion management. One of main purpose of our study was to dosimetrically evaluate how these two distinct approaches perform when subjected to unexpected respiratory variations. As our results in Figures 3 & 4 and Tables 3 & 4 demonstrate, these strategic differences led to significantly different dosimetric outcomes, and the reasons for the differences are explored in the discussion with Figure 6. To ensure this rationale is clear to all readers, we have added a paragraph in Discussion section clarifying why this comparison of different strategies is meaningful.

Line (285-293)

A direct comparison was made between ITV-SBRT and gated-SBRT, which were planned using different respiratory phase ranges (full phase for ITV and 30–70% respiratory phases for gating). This methodological difference is not a limitation but rather an intentional design that reflects the fundamental difference between two clinical strategies in lung SBRT motion management. The ITV-based approach is a ‘motion-encompassing’ strategy that aims to cover the entire range of tumor motion. In contrast, respiratory gating is a ‘motion-limiting’ strategy designed to minimize the irradiated volume by delivering the dose only during the most stable and reproducible portion of the respiratory cycle. Therefore, this comparison provides a direct evaluation of which distinct clinical strategy is more dosimetrically susceptible to unexpected respiratory motion changes.

3. Given the importance of dose accuracy in SBRT, particularly at target edges, could the authors discuss why 2.5 mm was selected for the calculation grid size, and whether any assessment was made of the potential impact compared to a finer resolution?

Thank you for this important point. We agree that finer grid sizes are generally recommended for SBRT where steep dose gradients are present. However, as numerous preliminary treatment plans had to be generated for this experiment, we chose 2.5mm as a reasonable balance between computational efficiency and accuracy. We believe that 2.5 mm calculation grid size for this study is acceptable for following reasons. First, the primary goal of this study was to evaluate relative dosimetric changes due to motion, not to validate absolute TPS accuracy. Also, published data indicate that the dosimetric difference from a finer grid is typically 1-2%, which is smaller than other physical uncertainties in our phantom experiment. To ensure clarity, we have added a justification for this choice in the Materials and Methods section of the manuscript.

Line (158-164)

While finer grid resolutions are often recommended for SBRT to accurately model steep dose gradients, this grid size was deemed appropriate for the specific objectives of this study. The goal of this study is to evaluate relative dosimetric changes due to motion, for which this resolution provides sufficient accuracy. Furthermore, previous studies have reported that the dosimetric differences between 2.5 mm grid to finer grid are generally within 1–2%, which is less significant thant the uncertainties introduced by respiratory motion and glass dosimeter measurements in current study[23, 24].

Reviewer #2:

The manuscript was well written and the data was organized well in its scope. Here are some comments and suggestions:

1. Since the comparison of SBRT plans was done using DCA technique, but not VMAT. It's better to reflect this in the title, as majority of the SBRT plans were made using VMAT now.

Thank you for your comment. We have revised the title and abstract as your recommendation.

2. The figures are really blurry and several labels can not be seen clearly.

We apologize for the inconvenience. The low resolution was likely an artifact of file compression during submission. We have replaced all figures with high resolution image files to ensure all labels and details are clear.

3. In line 30, as these are DCA plans, the plans were not really "optimized". Consider to use the phrase "were created".

Thank you for your comment. We have replaced “optimized” to “created” throughout the manuscript where it refers to DCA plans.

4. In line 36 and the following context, the term "upper and lower edges" were inaccurate. Please use "anterior and posterior edges" if it's in AP direction and "superior and inferior edges" if it's in SI direction.

Thank you for your comment. We have changed the term “upper and lower edges” to “superior and inferior edges” as your recommendation throughout the manuscript.

5. In line 134, it is mentioned the resolution size is 2.5mm, which is a little bit big for SBRT plan. Please include some explanation for this.

We agree that finer grid sizes are generally recommended for SBRT where steep dose gradients are present. As we explained, numerous preliminary treatment plans had to be generated for this experiment, we chose 2.5mm as a reasonable balance between computational efficiency and accuracy. We believe that 2.5 mm calculation grid size for this study is acceptable and we have added a justification for this choice in the Materials and Methods section of the manuscript.

Line (158-164)

While finer grid resolutions are often recommended for SBRT to accurately model steep dose gradients, this grid size was deemed appropriate for the specific objectives of this study. The goal of this study is to evaluate relative dosimetric changes due to motion, for which this resolution provides sufficient accuracy. Furthermore, previous studies have reported that the dosimetric differences between 2.5 mm grid to finer grid are generally within 1–2%, which is less significant than the uncertainties introduced by respiratory motion and glass dosimeter measurements in current study[23, 24].

6. In Figure 3 and Figure 4, there is no unit on y axis.

Thank you for your comment. We have corrected Figures 3 and 4 to include the appropriate units for y axis “Dose (cGy)”.

7. In line 178, Are the percentage values in the parenthesis the percentage of the dose delivered to these dosimeters in the plan. Please add some explanation to these values.

Thank you for your comment. The values are the percentages relative to the mean GTV dose for respective SBRT plans. While this was explained in the Materials and Methods section, we acknowledge that it was not clear in the context of the paragraph. We have revised the paragraphs with same issue to improve its clarity.

8. It would be clearer to include a table like table 2 to show what's the percentage dose change from the original values for different BPM and amplitude at different locations.

Thank you for your comment. We have added Table 3 and 4 to show the dose change from the original values for each respiratory condition and dosimeter location.
